# Plasmacytoid DC from Aged Mice Down-Regulate CD8 T Cell Responses by Inhibiting cDC Maturation after *Encephalitozoon cuniculi* Infection

**DOI:** 10.1371/journal.pone.0020838

**Published:** 2011-06-10

**Authors:** Jason P. Gigley, Imtiaz A. Khan

**Affiliations:** Department of Microbiology, Immunology and Tropical Medicine, George Washington University, Washington, D. C., United States of America; Karolinska Institutet, Sweden

## Abstract

Age associated impairment of immune function results in inefficient vaccination, tumor surveillance and increased severity of infections. Several alterations in adaptive immunity have been observed and recent studies report age related declines in innate immune responses to opportunistic pathogens including *Encephalitozoon cuniculi*. We previously demonstrated that conventional dendritic cells (cDC) from 9-month-old animals exhibit sub-optimal response to *E. cuniculi* infection, suggesting that age associated immune senescence begins earlier than expected. We focused this study on how age affects plasmacytoid DC (pDC) function. More specifically how aged pDC affect cDC function as we observed that the latter are the predominant activators of CD8 T cells during this infection. Our present study demonstrates that pDC from middle-aged mice (12 months) suppress young (8 week old) cDC driven CD8 T cell priming against *E. cuniculi* infection. The suppressive effect of pDC from older mice decreased maturation of young cDC via cell contact. Aged mouse pDC exhibited higher expression of PD-L1 and blockade of their interaction with cDC via this molecule restored cDC maturation and T cell priming. Furthermore, the PD-L1 dependent suppression of cDC T cell priming was restricted to effector function of antigen-specific CD8 T cells not their expansion. To the best of our knowledge, the data presented here is the first report highlighting a cell contact dependent, PD-L1 regulated, age associated defect in a DC subpopulation that results in a sub-optimal immune response against *E. cuniculi* infection. These results have broad implications for design of immunotherapeutic approaches to enhance immunity for aging populations.

## Introduction

Age related immune dysfunction includes decreases in generation of naïve CD4 and CD8 T cells, reduced thymic output, narrowing of TCR repertoires, changes in homeostatic proliferation and an accumulation of regulatory T cells [1], [2], [3], [4]. Recent studies have demonstrated that innate immunity, which is important for controlling early infection, is also defective with age as seen by decreased number and function of macrophages, neutrophils, NK cells and DC [5]. Furthermore, DC responses are down-regulated as a function of age [6], [7], [8] and since these cells are critical for both innate control of pathogens and the initiation of adaptive immune responses [9], dissecting the mechanisms behind their dysfunction as a result of this phenomenon are essential for adopting novel strategies to boost immunity in the elderly. Understanding the factors underlying decreased immune function with advancing age is important to help protect elderly populations against infectious disease and development of cancer.

DCs are important regulators of adaptive immunity and maintenance of tolerance [9] and can be generally divided into conventional DC (cDC) and plasmacytoid DC (pDC). DCs release cytokines which help skew immunity toward Th1, Th2, and Th17 responses [9]. Regulation of T cell responses by DC can be mediated by costimulatory molecule expression including the inhibitory molecule PD-L1, which is constitutively expressed by these cells [10]. Previous studies highlight that complex cDC-pDC and cDC-T cell interactions occur and are critical for generation of optimal immune responses [11], [12], [13]. Whether PD-L1/PD1 interactions between DC subsets play a role in regulation of T cell activation remains unknown.

Microsporidial infections (including *E. cuniculi*) are prevalent throughout the world [14], [15]. Microsporidia cause diarrheal disease as an opportunistic infection in HIV/AIDS patients and in non-HIV infected elderly populations [14], [16]. Understanding how age affects the immune response to *E. cuniculi* is important to reduce complications associated with this infection in aging individuals. During *E. cuniculi* infection, DC activation triggered via TLR4 and TLR2 dependent pathways results in IL-12 production resulting in robust CD4 and CD8 T cell dependent immunity which includes a potent intraepithelial lymphocyte (IEL) response [17], [18], [19], [20]. IFNg production by T cells and CTL activity are required for survival against *E. cuniculi* infection as mice lacking genes for this cytokine and perforin are both highly susceptible [17], [20]. Previous studies of *E. cuniculi* infection from our laboratory reported a defect in the DC response of 9–12 month old mice, suggesting that immune senescence begins earlier and the accumulating defects can have an impact on control of infectious pathogens as we age [21]. Supporting our hypothesis, recent studies with West Nile Virus (WNV) infections indicate that risk of developing meningoencephalitis after infection is greatly increased in middle aged (∼50 yrs) individuals [22], [23]. Recently, reduced pDC numbers were found to underlie decreased immunity in advanced age [24], [25] and that in late aged mice (18–26 months), pDC are reduced in their ability to produce IFNa in response to viral infection [26]. However, when these decreases in pDC function begin and how this impacts T cell activation in response to infection is not well elucidated. Furthermore, identifying novel mechanisms behind pDC-cDC crosstalk and how this interaction is modified with age have yet to be investigated. Although aged mouse pDC do not interact with young mouse cDC in an *in vivo* situation, this system was used to dissect the dysfunction of pDC from aged animals. In this study we demonstrate that pDC from aged animals have a cell intrinsic defect in their ability to mature and in a cell contact dependent mechanism via PD-L1 molecule suppress cDC maturation which leads to reduced CD8 T cell priming.

## Results

### Plasmacytoid DC response to *E. cuniculi* infection decreases as early as 12 months of age

**Figure 1:**
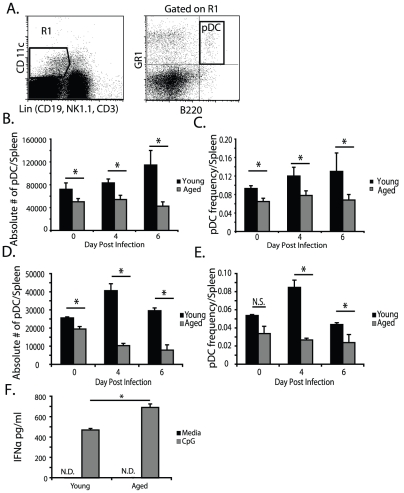
Decreased pDC abundance by 12 months of age after *E. cuniculi* infection. On day 4, and 6 post oral infection with 2×10^7^ *E. cuniculi* spores or 10 *Toxoplasma gondii* (ME49) cysts, splenocytes from 12 month aged or 8 week old young mice were harvested and analyzed by flow cytometry. Dot plot (A) depicts the gating stratey for pDC (CD11c lo-mid B220+ GR1+ Lin−). The absolute number (B,D) and frequency (C,E) of pDC per spleen at different time points after infection (Day 0 (naïve), 4 and 6) are shown in bar graphs for *E. cuniculi* (B,C) and *T. gondii* (D,E). For confirmation of pDC gating and sorting, sorted splenic pDC from young and aged mice were tested for their ability to produce IFNa after 36 hour stimulation with CpG DNA by ELISA. (F) A bar graph of IFNα production by sort purified pDC from aged or young mice is shown. Infection data (B,C, D, and E) shows mean ± SD from one experiment repeated 2–3 independent times with an n = 4 mice per group per experiment. IFNa data shown (F) is from 1 experiment repeated twice with similar results. *p≥0.05 by Student's *t* test.

Many studies investigating aging immune system dysfunction in mice utilize animals of advanced age (18 months or greater) [26], [27], [28]. We have previously published that age associated defects in DCs arise much earlier [21], however, whether pDC are also defective at an earlier age remains unknown. pDC are important for stimulating T cell responses to viruses and other pathogens including *T. gondii* [29], [30]. Therefore, we compared the absolute number and frequencies of pDC from young or 12 month old mice after oral infection with *E. cuniculi*. Single cell suspensions were made from spleens of infected animals and analyzed by flow cytometry for cell phenotype. As shown in figure 1, pDC which are CD11c^low-mid^B220^+^GR1^+^Lin^−^ (Fig. 1A) [31], [32] do not increase in absolute numbers and frequencies in spleens from aged mice in response to *E. cuniculi* compared to young mice (Fig. 1B and C). We found this lack of increase to also occur in response to infection with *T. gondii* (Fig. 1 D and E). pDC phenotype was verified using IFNa production by sorted pDC from aged and young animals in response to CpG stimulation (Fig. 1F). In sum, fewer numbers of pDC were observed in the spleens of 12-month-old as compared to young mice in response to *E. cuniculi* and *T. gondii* infection.

### pDC from aged mice suppress cDC ability to activate T cell response against *E. cuniculi*

**Figure 2:**
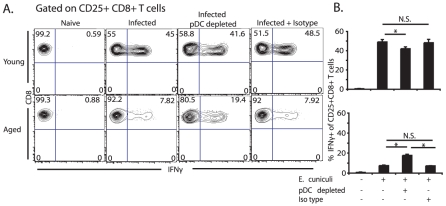
pDC depletion in aged mice increases CD8 T cell response to *E. cuniculi*. The level of CD8+ T cell IFNG production was measured in 12 month aged and 8 week old young mice in the absence of pDC. Both aged and young mice were depleted of pDC by i.p. injection of 150 µg/ml of 120G8 pDC specific antibody on day −1, 0, +1 and +3 post oral infection with 2×10^7^ *E. cuniculi* spores. Splenocytes were then harvest 10 days post infection and restimulated with irradiated *E. cuniculi* spores and surface stained for CD25 and CD8 then stained intracellularly for IFNg. (A) Contour plots show percent of activated CD25+ CD8+ T cells that are IFNg+in naïve (right), infected (middle right), pDC depleted infected (middle left) and isotype treated infected (left) young (top panels) or 12 M aged (bottom panels) mice. (B) Bar graph shows mean percent ± SD of CD25+ CD8+ T cells that are IFNg+. Contour plots are representative of 1 of 2 independently repeated experiments with an n = 4 mice per group per experiment. * denotes significance with p<0.05 by Student's *t* test.

As pDC are capable of activating T cells either by helping cDC or by acting alone after maturation [11], [29], [30], [33], [34], [35], [36], next, we measured how the functionality of these cells in either context is affected with age in response to both *E. cuniculi* infection. First, to determine the role of pDC in T cell activation *in vivo* we performed pDC depletion studies using the pDC specific 120G8 antibody and measured CD8 T cell responses in young mice. Figure S1 shows significant depletion of pDC as a result of antibody depletion. As shown in figure 2A and B, depletion of pDC from aged animals, significantly increased their CD8 T cell responses to *E. cuniculi* infection. Although pDC depletion in young mice resulted in a significant reduction in CD8 T cell activation (Fig. 2A and B), the differences were not overwhelming suggesting that pDC play a subordinate role in this process. Additionally, the recovery of CD8 T cell activation in aged animals did not reach levels measured in young mice and likely reflects additional age related defects in cDC and CD8 T cell function as previously reported [21], [37].

**Figure 3:**
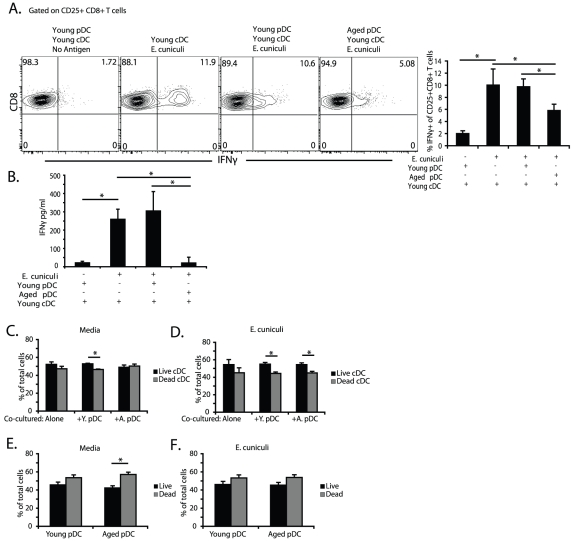
Aged pDC suppress CD8 T cell activation by cDC after *E. cuniculi* infection. To determine whether aged pDC could suppress T cell activation through their interaction with cDC we took 2×10^6^ sort purified splenic young cDC and cultured them alone or with either 4×10^5^ young or aged mouse splenic pDC. DC Cultures were then stimulated with irradiated *E. cuniculi* spores for 12 hours. 6 hours after DC depletion with DT, CD11c DTR EGFP mice received 5×10^5^ DC from the *in vitro* cultures. Four days later splenocytes from recipient mice were restimulated for 18 hours with irradiated *E. cuniculi* spores and activated CD25+ CD8+ T cell expression of IFNg was measured by intracellular staining. (A) Contour plots and bar graphs show the percent of CD25+ T cells that were CD8+ IFNg+. (B) Bar graph shows IFNg production from recipient mouse splenocytes cultured overnight as measured by ELISA. Separately, the viability of cDCs cultured alone, cDCs in co-culture with aged or young pDC or pDC alone was measured by flow cytometry before and after stimulation with *E. cuniculi* spores using fixable Live/Dead staining. (C,D) Bar graphs show percent of total live and dead cDCs before (C) or after (D) *E. cuniculi* stimulation. (E,F) Bar graphs show percent of total live and dead pDC before (E) and after (F) *E. cuniculi* stimulation. Contour plots are representative of 1 of 3 independent experiments repeated separately with an n = 4 mice per group per experiment. Bar graphs in A and B show mean ± SD. Bar graphs in C, D, E and F are representative of 1 of 3 individual experiments repeated independently and show mean ± SD. Statistical significance is indicated by * with p<0.05 by 1 way ANOVA and Bonferonni's multiple comparison test.

Since pDC have been shown to enhance cDC ability to prime CD8 T cell responses [33] we tested whether aged pDC could alter this process. To address this we co-cultured cells (cDC and pDC) from young and aged mice and subsequently used them for *in vivo* T cell activation assays. cDC were cultured alone or in combination with pDC from young or aged mice and then pulsed with *E. cuniculi* spores. These co-cultured cells were then adoptively transferred into CD11c DTR transgenic mouse that had been DC depleted 6 hours prior to transfer with an i.p. injection of 100 ng Diphtheria toxin (DT). Successful depletion of DC form DTR transgenic mice after DT treatment is shown in figure S2. The level of T cell activation in the recipient animals was measured by assaying the CD25+ CD8+ T cell population for the frequency of IFNg+ cells by flow cytometry. As in agreement with previous reports, DT treated CD11c DTR mice did not survive longer than 5 days, the assays were performed 4 days after DT injection [38]. Treatment of recipient mice with young cDC alone or young cDC-young pDC co-cultures induced significant number of CD8+ T cells expressing IFNg+ (Fig. 3A). No significant difference was observed in CD8 T cell activation between recipients injected with young cDC alone or young pDC-cDC co-cultures (Fig. 3A, bar graph). Conversely, CD8 T cell responses against *E. cuniculi* in animals receiving young mouse cDC-aged mouse pDC co-cultures were significantly lower (Fig. 3A). Additionally, IFNg, measured in the supernatants from total splenocyte restimulation by ELISA, validated the intracellular staining (Fig. 3B). To determine whether the difference observed in CD8+ T cell activation was due to cDC death, we assayed the viability of cDC cultured alone or in co-culture with aged or young mouse pDC before and after stimulation using Live/Dead staining. Live cells react minimally with the fluorescent dye on their surface and stain dimly while dead cells with compromised membranes highly react with the dye and become brightly stained. There was no significant difference in viability between the different culture conditions (Fig. 3C and D) or between aged or young mouse pDC (Fig. 3E and F) after *in vitro* stimulation with *E. cuniculi*.. Therefore, these data suggested that the majority of *in vivo* CD8 T cell activation in response to *E. cuniculi* infection was cDC dependent and that aged mouse pDC are capable of suppressing cDC ability to efficiently activate the CD8 T cell response. This suppression was not correlated with a decrease in cDC or pDC viability.

### pDC from aged mice suppress the ability of cDC to activate T cells via cell contact

**Figure 4:**
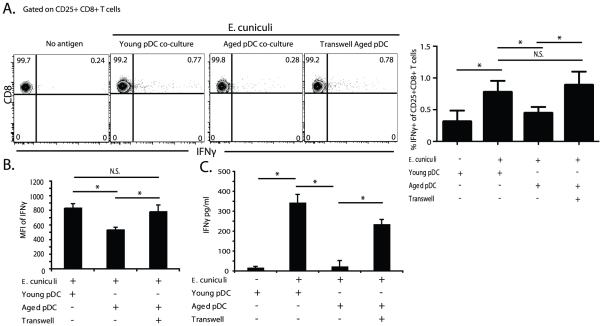
Aged pDC suppress cDC ability to prime CD8 T cells via cell contact. To determine whether aged pDC suppressed cDC ability to prime CD8 T cells in a cell contact dependent manner co-cultures were established in transwell culture plates. 2.0×10^6^ sort purified young (8 week) splenic cDC were co-cultured with 4×10^5^ young or aged (12 month) splenic pDC in cell contact or kept separated by a permeable membrane in the upper chamber of a transwell culture system. Co-cultures and each chamber of the transwell culture system were then stimulated at a 1∶1 ratio with irradiated *E. cuniculi* spores for 12 hours. After stimulation cDC were re-sorted to remove pDC and extracellular *E.cuniculi* spores. Subsequently, 2.5×10^5^ young cDC were transferred to DC depleted CD11c DTR transgenic mice (DT 100 ng 6 hours). Four days later splenocytes of recipient mice were harvested, restimulated for 18 hours with *E. cuniculi* and assayed for the percent of activated CD25+ CD8+ T cells expressing IFNg+ by flow cytometry. (A) Representative contour plots show frequency of activated CD25+ CD8+ T cells that are IFNg+ in splenocytes from recipient mice. (A) Bar graphs of the percent IFNg+ of CD25+CD8+ T cells show mean ± SD from 2 separate experiments. (B) Bar graphs show the mean ± SD MFI of IFNg in activated CD25+ CD8+ T cells of recipient mice. Data in bar graphs in A and B were analyzed by 1 way ANOVA and Bonferroni's multiple comparison test with * p<0.05. Separately, recipient mouse splenocytes were re-stimulated overnight and IFNg in the supernatants measured by ELISA. (C) Bar graph shows mean ± SD of IFNg production by restimulated recipient mouse splenocytes. Data is representative of 2 experiments repeated separately with an n = 4 mice/group. *p<0.05 by Student's *t* test.

Previous studies have reported direct pDC and cDC interactions *in vivo* which can lead to optimal T cell activation [11], [33]. Given our results (shown in figure 3) from co-culturing pDC and cDC, we tested whether or not the suppression of cDC function by pDC from aged mice was cell contact dependent. To determine this, co-cultures of mixed DC populations (cDC and pDC) were established in cell contact or in a transwell system where cells are separated by a membrane. After 12 hours of *E. cuniculi* stimulation, cDC from all co-cultures were re-sorted and subsequently transferred to DC depleted CD11c-DTR transgenic mice. Resorting of cDC was performed to provide additional control for any possible effect, as previously suggested, pDC may have on *in vivo* T cell priming [30], [34]. The recipient mice were then assayed for CD8 T cell activation 4 days after cell transfer. As shown in figure 4A, when co-cultured with pDC from aged mice, ability of cDC from young animals to prime *in vivo* CD8 T cell responses was significantly reduced compared to when the cells were co-cultured with pDC from young mice. However, when pDC from aged animals were separated from cDC using a transwell during antigen pulse, priming of CD8 T cells to produce IFNG was restored (Fig. 4A). Moreover, the mean fluorescent intensity (MFI) of CD8+ T cell IFNg and IFNg production, as measured by ELISA from supernatants of restimulated splenocytes, was significantly reduced in mice that received young cDC co-cultured in cell contact with aged mouse pDC (Fig. 4B and C). Although transferred resorted cDC induced a CD8 T cell response, we observed significantly lower CD8 T cell activation than data shown in figure 3. This data likely reflects the reduced number of cDC transferred (2.5×10^5^ in this figure vs. 5×10^5^ in figure 3). Figure S3A and B show a dose titration of resorted cDC resulted in decreasing frequency of CD8+ IFNg + T cells in recipient mice. These data also reflect that the resorting process decreased cell viability by 50% (Fig. S3D). Interestingly, the dose titration did not result in reduced MFI of CD8+ T cell IFNg (Fig. S3C). Reduced MFI of CD8+ T cell IFNg only occurred when cDC were co-cultured with aged mouse pDC in cell contact suggesting that the reduced quality of CD8+ T cell activating signal was cell contact dependent. Taken together these findings suggest that in aged animals, pDC exhibit a cell contact dependent suppression of cDC and cause a down regulation of CD8 T cell activation *in vivo*.

### pDC from aged mice express lower costimulatory molecules and higher PD-L1 than pDC from young mice in response to *E. cuniculi* infection

**Figure 5:**
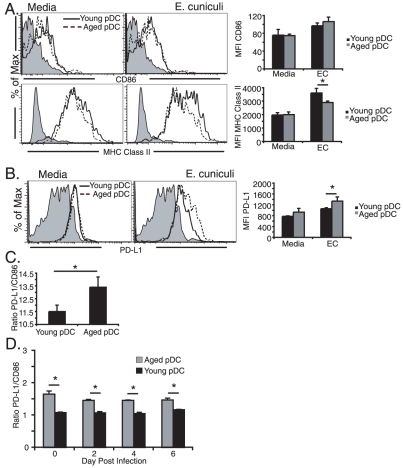
pDC from aged mice express higher PD-L1 than CD86. Sort purified pDC from naïve young and aged mice were plated and cultured in the presence of irradiated *E. cuniculi* spores and 12 hours later analyzed for maturation. (A) Histograms for (top panels) CD86 and (bottom panels) MHC Class II are shown with mean MFI values. Corresponding bar graphs show the mean ± SD MFI for each marker measured. (B) Histograms for PD-L1 expression by pDC after *E. cuniculi* stimulation are shown with corresponding bar graph (left) of mean MFI ± SD. (C) Bar graph shows PD-L1/CD86 ratio on young or aged pDC after *in vitro* stimulation with *E. cuniculi*. Separately, young or aged mice were inoculated orally with *E. cuniculi* and splenic pDC assayed *ex vivo* for PD-L1 and CD86 expression by flow cytometry on days 0, 2, 4 and 6 post infection. (D) Bar graph shows mean ± SD of the PD-L1 to CD86 ratio on pDC from infected mice. Experimental data presented was repeated independently twice. *Ex vivo* experiments had an n = 4 mice per group. * denotes significance with p<0.05 by Student's *t* test.

Lower expression of MHC class II, CD86, CD80 and CD40 by DC can lead to T cell tolerance and anergy resulting in lower naïve T cell activation [39]. High level expression of the recently described down regulatory molecule PD-L1 results in decreased T cell activation [10] especially when there is a higher ratio of PD-L1 to CD86 on pDC [40]. Since pDC from 12-month-old mice exhibited normal IFNα production and did not differ in transcription of IDO (data not shown) compared to young cells, next, we investigated if the defect was related to a difference in their expression of these molecules following *E. cuniculi* infection. Therefore, splenic pDC were sort purified, subsequently stimulated *in vitro* with *E. cuniculi* spores for 12 hours and assayed for costimulatory molecule expression. We did not observe significant differences in the levels of CD40 or CD80 (data not shown), however, the level of CD86 expression on both aged and young pDC increased similarly with stimulation (Fig. 5A top panels). Interestingly, MHC class II expression on the cells from older animals remained lower (Fig. 5A, bottom right panel). Moreover, pDC from aged animals expressed PD-L1 at higher levels after stimulation than young pDC (Fig. 5B) and this molecule was found to be at a higher ratio than CD86 (Fig. 5C). Next, to verify our *in vitro* data, we measured the PD-L1/CD86 ratio *in vivo* after *E. cuniculi* infection in young and aged mice. The ratio of PD-L1 to CD86 was higher for pDC from aged mice compared to cells from young animals at all time points tested, suggestive of a more regulatory phenotype (Fig. 5D). Differences in expression of ICOSL and GITRL between pDC of young or aged animals were not noted (data not shown). These data suggest that pDC from older mice have reduced expression of CD86 and MHC Class II than PD-L1 resulting in a more tolerogenic phenotype.

### pDC from aged mice inhibit cDC maturation in cell contact dependent manner

**Figure 6:**
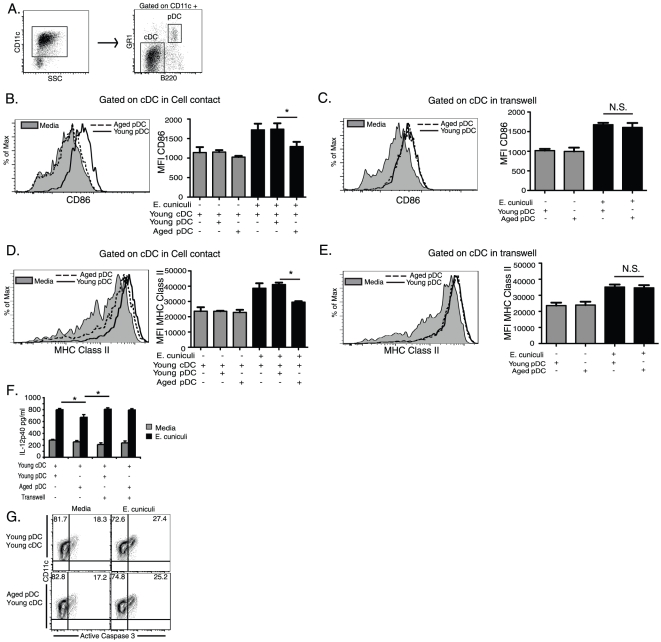
pDC from aged mice suppress cDC maturation via cell contact. To test cell contact dependence of aged pDC effect, 1.25×10^5^ sort purified naïve splenic young (8 week) mouse cDC were co-cultured with or without 1.0×10^4^ naïve young or aged (12 month) mouse splenic pDC or separated by a permeable membrane in a transwell culture system then stimulated 1∶1 with irradiated *E. cuniculi* spores for 12 hours. After live/dead staining and exclusion of cell clusters as shown in (A) cDC (CD11c hi B220-GR1−) were gated and analyzed for (B and C) CD86 and (D and E) MHC Class II in cell contact (B and D) or in transwell (C and E) cultures. Representative histograms show expression of CD86 (B and C) and MHC Class II (D and E) by cDC co-cultured in cell contact (B and D) or separated by a transwell (C and E) from aged pDC (dashed line) or young pDC (solid line) before (gray filled histogram) or after stimulation with *E.cuniculi* spores. Bar graphs show mean ± SD MFIs of CD86 (B and C) and MHC Class II (D and E) on cDC from cell contact (B and D) or transwell (C and E) cultures conditions. Bar graphs for CD86 (B) and MHC Class II (D) also included cDC cultured alone with and without stimulation. Data presented in bar graphs is from 2 independently repeated experiments. * denotes significance measured by 1 way ANOVA and Bonferroni's multiple comparison test with p<0.05. (F) Bar graph shows IL-12p40 production from supernatants of pDC and cDC co cultures (* denotes significance with p<0.05 by Student's *t* test). Separately, to measure level of cDC apoptosis, co-cultured cDC were gated as in A and assayed for active caspase-3 staining by flow cytometry. (G) Contour plots show percent CD11c+ active caspase-3+positive cells. This experiment was repeated independently twice in triplicate with similar results. Data presented is from 1 of those experiments.

Given that cDC driven CD8 T cell activation was decreased when cultured with aged pDC in a cell contact dependent mechanism (Fig. 3), we next investigated how aged pDC could modify cDC function. We first evaluated the ability of aged or young mouse cDC to express costimulatory molecules and MHC Class II in response to *E. cuniculi* infection. Differences between aged and young cDC in response to *E. cuniculi* stimulation were observed for CD86 and MHC Class II but no other significant differences were observed for other costimulatory molecules tested (CD80, CD40, CD40l, PD-L1 and 2, GITRL and ICOSL) (data not shown). Therefore these markers were used as a measure of maturation of the cDC. Expression of CD86 and MHC Class II by aged cDC was significantly reduced (Fig. S4A–D) as compared to young cells post *E. cuniculi* stimulation, even in the presence of young pDC. Given this apparent inherent defect in aged cDC, we decided to continue this study with functionally intact young mouse cDC to assay the aged mouse pDC suppression of this cell type. We then determined whether aged mouse pDC could alter cDC maturation by measuring their level of CD86 and MHC Class II expression in response to *E. cuniculi* infection. Mixed pDC-cDC co-cultures with and without cell contact using a transwell were established as described above and maturation of the cDC (gated as shown in Fig. 6A) was compared after stimulation with *E. cuniculi* spores. CD86 and MHC Class II expression on cDC cultured alone or from co-cultures with pDC from young mice were increased regardless of cell contact (Fig. 6B and D). However, when co-cultured in cell contact with pDC from aged mice, the expression of CD86 and MHC Class II (Fig. 6B and D) was lower on the young cDC than controls. No significant difference was observed when aged mouse pDC were in contact with aged mouse cDC (Fig. S4E). Importantly, when pDC from aged mice were separated from young cDC by a transwell, the decreased levels of CD86 and MHC Class II expression observed in cell-contact cultures were recovered and not significantly different from young mouse controls (Fig. 6C and E). To determine whether other cDC functions were affected by aged pDC, we measured IL-12p40 in supernatants from the co-cultures. As shown in figure 6F, IL-12p40 production, an important inflammatory cytokine produced by DC [41] was only modestly reduced in aged pDC:young cDC co cultures. To further define the mechanism of cell contact dependent aged pDC suppression we determined whether cDC in contact with aged pDC undergo apoptosis by measuring levels of the pro-apoptotic molecule active caspase-3. Interestingly, we found no difference in active caspase-3 levels between young or aged pDC:young cDC co cultures (Fig. 6G). These results suggest that the reduced ability of cDC to prime CD8 T cells caused by pDC from aged mice was dependent on a cell contact mediated decrease in cDC maturation and not attributed to an increase in apoptosis.

### pDC from aged mice suppress cDC ability to mature via a PD-L1 dependent mechanism

**Figure 7:**
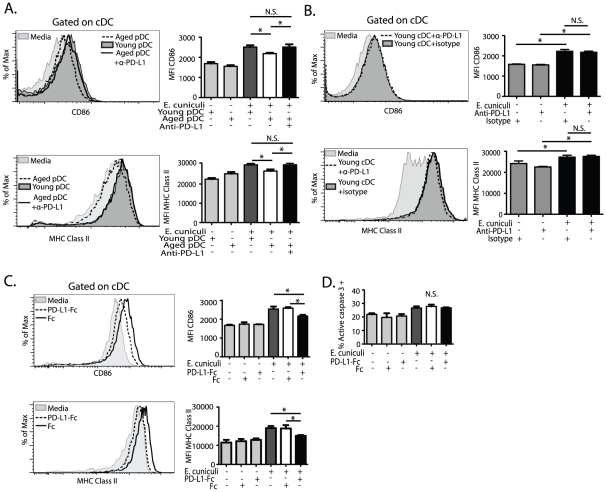
Aged pDC suppress cDC maturation via PD-L1 dependent pathway. 1.25×10^5^ sorted naïve splenic young cDC were co-cultured with 1.0×10^4^ naïve splenic young or aged mouse pDC with anti-PD-L1 (30 µg/ml) then stimulated with irradiated *E. cuniculi* spores for 12 hours. (A) Representative histogram shows CD86 (top panel) and MHC Class II (bottom panel) expression on unstimulated (light gray filled) cDC or on cDC stimulated with *E. cuniculi* while in co-culture with aged pDC (dashed line), young pDC (dark gray filled) or aged pDC with anti-PD-L1 antibody (solid line). (B) Representative histograms show CD86 (top panel) and MHC Class II (bottom panel) expression on cDC cultured alone without (light gray filled) or with *E. cuniculi* stimulation in the presence of either anti-PD-L1 antibody(dashed line) or isotype control (dark gray filled). (A and B) Bar graphs show mean MFI ± SD of CD86 (top panels) and MHC Class II (bottom panels) on young cDC co-cultured with pDC (A) or culture alone (B) before (light gray bars) and after *E. cuniculi* stimulation with the following conditions; young pDC co-culture (dark gray bar), aged pDC co-culture (white bar) and aged pDC co-culture with anti-PD-L1 (black bars). (C) To confirm that PD-L1 can alter cDC maturation, cDC were cultured in the presence of plate bound PD-L1 Fc fusion protein (30 µg/ml), control Fc or no treatment then stimulated with *E. cuniculi* spores. (C) Representative histograms and bar graphs of mean ± SD MFI for CD86 (top panel) and MHC Class II (bottom panel) are shown. (D) Bar graph shows percent of cDC active caspase-3 positive ± treatments shown in C. Data presented are from 2 experiments repeated independently with quadruplicate replicates.* denotes significance with p<0.05 by 1 way ANOVA and Bonferonni's multiple comparison test.

PD-1 engagement on DCs by PD-L1 has been reported to cause down regulation of their IL12 production during infection [42]. Also PD-L1 expression on DC sub-populations has been reported to suppress allogeneic responses and induce tolerance [40], [43]. Since we observe that pDC from aged animals via contact with cDC can reduce their ability to mature and prime T cell response, involvement of this molecule in this process was examined. Splenic DC sub-populations were sorted, co-cultured with blocking anti-PD-L1 antibody, stimulated with *E. cuniculi* and expression of CD86 and MHC Class II on cDC measured. As shown in Fig. 7A (black bars), addition of anti PD-L1 antibody to co-cultures (aged pDC-young cDC co-cultures) restored CD86 and MHC Class II expression on cDC to normal levels. Conversely, anti-PD-L1 or isotype control treatment of cDC alone (Fig. 7B) did not enhance CD86 and MHC Class II expression prior to or after stimulation further supporting that only aged mouse pDC are able to suppress via their PD-L1 expression. The result obtained with the isotype control (Fig. 7B) agrees with a previously published report and in accordance with this previous study, we excluded this control from future experiments due to the large number of mice needed to obtain enough cells for each experiment [44]. To further establish the affect of PD-L1 directly on cDC we performed assays using plate bound PD-L1-Fc fusion protein. PD-L1-Fc fusion protein decreased young cDC ability to express CD86 and MHC Class II (Fig. 7C) compared to cultures treated with Fc protein alone or no treatment without increasing apoptosis as measured by cDC expression of active caspase-3 (Fig. 7D). These results demonstrate that pDC from aged animals can down-regulate cDC ability to mature through a PD-L1 dependent mechanism.

### PD-L1 suppression mediated by pDC from aged animals does not affect expansion but function of antigen specific CD8 T cells

**Figure 8:**
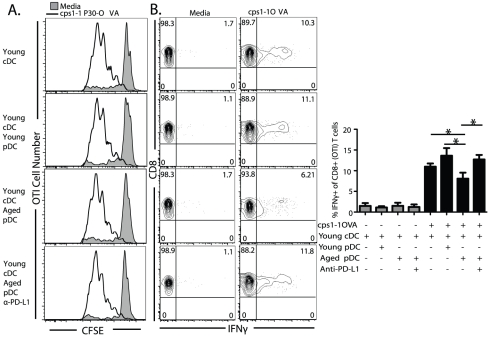
PD-L1 mediated suppression of effector function not expansion of antigen specific T cells by aged mouse pDC. Sorted young cDC with or without young or aged mouse pDC were stimulated with RHΔ*cps1-1*P30-OVA transgenic tachyzoites for 12 hours in presence or absence of anti-PD-L1 (30 µg/ml). For proliferation, 2×10^4^ CFSE labeled CD8+ OTI T cells and 5×10^5^ CD45.1 TCRb purified T cells were added to the cultures and 72 hours later OTI cells were analyzed for (A) CFSE dilution shown by histogram. To assess effector function, 2×10^3^ CD8+ OTI T cells and 5×10^5^ CD45.1 TCRb purified T cells were added to the cultures and after 3 days restimulated with freshly isolated CD45.1 splenocytes and 1 µg/ml SIINFEKL peptide for 5 hours then intracellularly stained for IFNG. (B) Contour plots show OTI IFNg production and frequencies. (B) Bar graph shows mean ± SD of percent of IFNg+ OTI T cells. * denotes significance with p<0.05 by 1 way ANOVA and Bonferroni's multiple comparison test. Data is representative of one experiment repeated independently twice in quadruplicate.

Relevant tools including TCR transgenic mice and identification of dominant CD8 T cell epitopes to study antigen specific T cell priming in response to *E. cuniculi* infection have yet to be developed. Additionally, whether this age dependent mechanism of immune suppression occurs with other infections has not been studied. Therefore to broaden the implications of our study and to determine whether the findings can be extended to other infections, we took advantage of the OTI TCR transgenic system by using ovalbumin-expressing tachyzoites of *Toxoplasma gondii* (*cps1-1*P30-OVA). DC co-cultures were established and pulsed with *cps1-1*P30-OVA tachyzoites with and without anti-PD-L1 treatment. To assay for proliferation, CFSE labeled OTI CD8 T cells were added to cultures with total T cells from congenic CD45.1 mice, used as an internal negative control for non-antigen specific polyclonal response, and CFSE dilution was measured 72 hours later. Interestingly, young cDC driven OTI expansion was not affected by co-culture with pDC from aged mice with or without anti-PD-L1 treatment (Fig. 8A). Therefore, we assayed whether effector function of the OTI cells was decreased, by measuring their intracellular IFNg production. Co-culturing cDC with pDC from young animals did not significantly alter the level of OTI+IFNg+ T cells (Fig. 8B). Similar to data presented in figures 2 and 3, cDC co-cultured with pDC from aged mice and stimulated with *cps1-1*P30-OVA significantly reduced the percentage of IFNg+ OTI CD8 T cells (Fig. 8B). As shown in Fig. 8B, addition of anti-PD-L1 to aged mouse pDC–young cDC cultures restored the level of OTI IFNG production to levels similar to control cultures. Anti-PD-L1 treatment of cDC cultures alone did not have any effect on the level of OTI IFNg production (data not shown). These data strengthen our results obtained with *E. cuniculi* infection and reveal that pDC from aged mice suppress cDC priming of T cells in an antigen specific manner through a PD-L1 dependent mechanism. The data presented here strongly suggest that pDC could contribute to decreased immunity with aging at a younger than previously thought age through the conditioning of cDC *in vivo* at sites of inflammation.

## Discussion

Microsporidial infections of humans (including *Encephalitozoon cuniculi*) occur worldwide with prevalence rates of up to 50% depending on geographic region, method of diagnosis, socioeconomic and immune status of the population studied [14], [15]. Microsporidial infections have been reported to be a common cause of diarrheal disease and create complications in non-HIV infected elderly populations [16]. Previous studies of *Encephalitozoon cuniculi* infection from our laboratory reported a defect in the DC response of 9–12 month old mice, suggesting that immune senescence begins earlier and the accumulating defects can have an impact on control of infectious pathogens as we age [21]. Supporting our hypothesis, recent studies with WNV infections indicate that risk of developing meningoencephalitis after infection is greatly increased in middle aged (∼50 yrs) individuals [22], [23]. By adding the *T. gondii* model in this study and observing defects in murine DC responses to this parasite at 12 months of age, we further support and expand the middle-aged immune senescence hypothesis to a broad range of pathogens.

Recent investigations of DC subpopulation biology in response to infectious pathogens have revealed that pDC are capable of interacting with cDC and help them mature resulting in optimal CD8 T cell activation and function [11], [33]. In the current study, pDC biology as a function of age was further dissected by investigating a) whether they phenotypically and functionally decline, b) if age associated pDC dysfunction could alter CD8 T cell activation and c) whether pDC from aged animals cause reduced CD8 T cell activation through their interaction with cDC. We observed that compared to younger mice, spleens of middle-aged animals contained fewer pDC in response to *E. cuniculi* and *T. gondii* infection. We also observed that *in vivo* depletion of pDC from 12 month aged mice partially restored CD8 T cell priming only in these animals not in young mice. Moreover, when co-cultured with pDC from aging mice, the ability of young cDC to prime CD8 T cell responses against *E. cuniculi* was decreased. Interestingly, we further demonstrate that this suppression was not mediated by pDC production of the T cell suppressive enzyme IDO. The aged pDC dependent decrease in cDC ability to prime CD8 T cells was cell contact dependent and driven by the high expression of the inhibitory molecule, PD-L1. This down-regulation of cDC ability to prime T cells mediated by pDC from aged animals was not unique to *E. cuniculi* infection and was also observed in an antigen specific manner with *Toxoplasma gondii*. Similar to *E. cuniculi* infection, the suppressive effect of pDC on T cell immune response against *T. gondii* could be neutralized by anti-PD-L1 antibody treatment. These observations highlight the intricate relationship between pDC and cDC and the downstream effect on T cell priming and for the first time suggests that pDC-cDC crosstalk is a potential mechanism by which immune responses to *Encephalitozoon cuniculi* and other pathogens may be decreased with advancing age.

pDC are recruited to draining lymph nodes and spleens via the high endothelial venule in a CXCL9 and CD62L dependent manner [45]. The decrease in frequency and number of pDC we observed after infection with *E. cuniculi* could be a result of a decreased ability of these cells to traffic as has been reported for cDC in advanced age [8]. However, several studies in elderly humans report that pre-pDC frequency decreases in the periphery suggesting that similar phenomenon may be occurring in mice [24], [46]. pDC can arise from either common myeloid progenitors (CMP) or common lymphoid progenitors (CLP) and it is possible that the decrease in frequency of pDC recruited to sites of inflammation with age could be a result of reduced CLP output [47]. Another possibility could be that increases in monocyte generation observed in aged bone marrow from enhanced CMP differentiation may be suppressing the differentiation of the different subpopulations of DCs or that pDC differentiation may be halted in the monocyte phase in a homeostatic manner. Although a decrease in pDC numbers occurred, we find that the pDC present during infection of aged mice are more suppressive, as observed after pDC depletion in aged mice CD8 T cell responses are partially recovered (Fig. 2). This suggests that the decrease in pDC numbers could reflect reduced recruitment of a more inflammatory subpopulation of pDC, and maintenance of a tolerogenic pDC population which express CCR9 and CD19 [48], [49]. However, we did not observe higher levels of IDO transcription by the pDC in aged mice, reported to be an important function of tolerogenic pDC and our gating strategy excludes CD19+ pDC.

In the current study we report that in addition to their decreased number, pDC from aged mice inhibit the maturation of cDC needed for T cell activation in response to pathogen. The interaction of pDC with cDC may be a critical mechanism of optimal cDC maturation and activation of a T cell response [11]. pDC are recruited from the periphery to sites of inflammation through the HEV, therefore it is possible that co-localization of pDC, cDC and T cells could lead to an increase in their interactions and crosstalk [31], [45]. Activated T cells are known to further mature cDC via their CD40L interaction with CD40 on the DC [12], [41]. Recently, pDC were implicated to help LN DC efficiently induce CTL responses via a CD40-CD40L dependent pathway [33]. Although we do not directly measure the level of pDC-cDC interaction *in vivo*, pDC depletion from aged mice results in partial restoration of CD8 T cell responses to *E. cuniculi*, which suggests that with age they acquire a more tolerogenic phenotype. One mechanism by which this could occur is through crosstalk with cDC as previously established in other models of infection [11], [33]. Our study confirms the occurrence of this crosstalk by showing that aged pDC can down-regulate cDC maturation *in vitro* via a cell contact dependent mechanism. Further dissection of this mechanism will be interesting for future studies. Although CD40-CD40L interactions are important for pDC-cDC interaction, based on our results, we hypothesize that this is independent of CD40-CD40L interaction as we did not find differences in CD40L expression between young or aged mouse pDC (data not shown) [11], [33].

Expression of PD-L1 (a recently described down regulatory molecule) on APCs can cause an increase in tolerance and is associated with CD8 memory T cell dysfunction in chronic viral infections [50], [51]. Data presented herein show pDC-cDC crosstalk via PD-L1 dependent mechanism resulting in decreased maturation of cDC by aged pDC. Blockade of PD-L1 with anti-PD-L1 antibody restored the maturation of cDC to those of young pDC-cDC co-cultures and rescues the antigen specific CD8 T cell effector function downstream of cDC activation. These findings are supported by studies with *Listeria monocytogenes* showing that increased PD1 (a receptor for PD-L1) expression on DC down regulates their production of IL-12 and use of PD1^−/−^ DC restores function and innate immunity to bacterial infection [42]. Moreover, it has been demonstrated, that expression of CTLA-4 on T cells can decrease CD80 and CD86 expression on maturing DC [52] suggesting that cDC interaction with different cell types can modulate the level of DC maturation. Although we did not find decreased CD80 and CD86 expression on un-stimulated cDC co-cultured with pDC from aged animals, ability of cDC to mature could be suppressed after activation of the cDC via the higher expression of PD-L1 on aged pDC [52]. Interestingly, we also find that this decreased level of maturation is not a result of increased apoptosis as measured by active caspase-3 (Fig. 6 and 7). Elucidating the downstream affects of PD-L1 expression in pDC from aged animals on the molecular process of cDC maturation warrants further study. However, blockade of PD-L1 engagement with the receptor may be a potential therapeutic approach to enhancing vaccine responses in the elderly.

Overall in the present study we report that by 12 months of age in mice, pDC numbers are decreased and these cells compromise the ability of young cDC to activate CD8 T cells. The suppressive phenotype of aged pDC seems independent of soluble factors and is caused by cell contact via a PD-L1 mediated down regulation of cDC ability to mature. This pDC derived PD-L1 driven decrease in cDC maturation ultimately results in decreased antigen specific CD8 T cell effector function and represents a potential mechanism by which immune senescence could occur in the elderly. This mechanism may be a reason why *Encephalitozoon cuniculi* causes increased diarrheal disease and complications in the non-HIV infected elderly individuals. Fully dissecting the cellular and molecular events leading to this suppressive effect of aged pDC will allow for better design of vaccine strategies to enhance immunity and decrease morbidity and mortality associated with infection in aging populations.

## Materials and Methods

### Mice, parasites and infection

12 month aged and young (6–8 wks) C57BL/6 mice were purchased from Charles River (Germantown, MD), CD11c DTR/EGFP mice, OTI TCR transgenic and CD45.1 congenic C57BL/6 mice were purchased from Jackson laboratories (Bar Harbor ME). All Animals were maintained in the animal resource facility at George Washington University Medical Center (Washington, DC), according to institutional IACUC approved protocols. All experiments were approved by the American Association for Accreditation of Lab Animal Care (AAALAC) certified George Washington University Medical Center Animal Resource Facility Institutional Animal Care and Use Committee (IACUC) under the protocol approval number of A053. *Encephalitozoon cuniculi* spores, *Toxoplasma gondii* cysts (ME49) and tachyzoites of strain RH Δ*cps1-1*-P30-OVA (F. Dzierszinski McGill, Montreal, Canada), were maintained as previously described [21], [53]. For all *in vivo* experiments, mice were infected orally.

### Plasmacytoid and conventional DC isolation, purification and phenotyping

pDC and cDC were obtained from the MLN and spleen of aged and young mice following a previously published protocol [54]. Briefly, single cell suspensions were made from mechanically disrupted Collagenase D (SIGMA) digested organs. For all experiments anti-CD11c biotin positively selected cells were sort purified into pDC and cDC (FACSAria, BD Biosciences) to >98% using antibodies to B220, Lin- (CD19, CD3, NK1.1) and GR1 (eBioscience) as previously described [31], [55].

### Antigen specific *in vitro* T cell priming

5×10^4^ cDC were co-cultured with 3×10^3^ pDC from either young or aged animals and pulsed overnight at a previously described ratio [29] 1∶1 with RHΔ*cps1-1*P30-OVA tachyzoites with or without anti-PD-L1 (9G2, 30 ug/ml BioXcell). For proliferation assays 5×10^5^ CD45.1 T cells (used as an internal negative control for non-antigen specific polyclonal responses) and 2×10^4^ purified 10 µM CFSE (Invitrogen) labeled OTI CD8 T cells were added to each well of DC co-culture. After 72 hours, expansion was measured by flow cytometry (CFSE Dilution, FACScalibur, BD Biosciences). To assay OTI effector function, DCs cultures were set up as described above with 2×10^3^ purified OTI CD8 T and after 72 hours restimulated with fresh CD45.1 total splenocytes, 1 ug/ml SIINFEKL peptide (Anaspec) and 1× Brefeldin A/Monensin (BD Biosciences) for 5 hours. OTI specific intracellular IFNg was then measured by flow cytometry.

### *In vivo* DC reconstitution, T cell priming and pDC depletion

2×10^6^ cDC from young mice were plated in 24 well and co-cultured with 4.0×10^5^ pDC isolated from young or aged animals. Co-cultures in one well or both sides of transwells were stimulated at a previously described ratio [21], 1∶1, overnight with irradiated *E. cuniculi* spores. Subsequently, either 5×10^5^ total cells from co-cultures or 2.5×10^5^ resorted cDC were injected into DC depleted (100 ng Diphtheria toxin (SIGMA), 6 hours) CD11c DTR-EGFP mice via intravenous (i.v.) route. Four days later, recipient mouse splenocytes were prepared and restimulated with irradiated *E. cuniculi* spores. After a 12-hour incubation, the cells were treated with 1× Brefeldin A and 1×Monensin for 6 hours. CD25+ T cells were analyzed for CD8 and IFNg by flow cytometry. For *in vivo* pDC depletion, mice were injected i.p. with 150 ug of 120G8 monoclonal antibody or isotype control (Rat IgG, Jackson Immunoresearch) on day −1,0, +1 and +3 post infection as described [56].

### Transwell DC stimulation for *in vivo* and *in vitro* assays

For *in vivo* DC reconstitution assays 2×10^6^ sort purified cDC form young mice were plated in the bottom chamber of a 24 well transwell plate (0.4 µm pore size, Corning). To the top chamber, 4×10^5^ aged or young pDC were added. Co-cultures in the transwell plates were then stimulated at a 1∶1 ratio of DC∶*E. cuniculi* spores in both chambers of the transwell. 12 hours later the cells were harvested for resorting as described above. For *in vitro* cell contact dependent cDC maturation assays, 1.25×10^5^ cDC were plated into the bottom chamber of 96 well transwell plates (0.4 µm pore size, Corning). To the top chamber of each well containing cDC, 1.0×10^4^ pDC from aged or young mice were added. Both top and bottom chambers were then stimulated 1∶1 with *E. cuniculi* spores for 12 hours then cDC assayed for maturation by flow cytometry.

### *In vitro* and *in vivo* DC stimulation

Splenocytes from young and aged mice were harvested on day 0, 2, 4, and 6 p.i. as described above then assayed for maturation using 7-color flow cytometric analysis. For *in vitro* co-stimulation experiments, purified DC sub-populations were isolated and cDC co-cultured alone, pDC alone, or cDC with pDC from young or aged mice and with or without anti-PD-L1 (30 ug/ml, BioXcell) in either 96 well tissue culture plates. The cultures were then stimulated for 12 hours with irradiated *E. cuniculi* spores and assayed for maturation by flow cytometry. For cDC alone maturation inhibition assays, 96 well tissue culture plates were coated in sterile conditions with PD-L1 fused to human Fc (PD-L1 Fc) or human Fc (Fc) (R&D Systems) at 100 µg/ml in 1× PBS overnight at 4°C. Plates were then washed 3 times with sterile 1× PBS. 1.25×10^5^ sort purified cDC were then added to each well and stimulated with irradiated *E. cuniculi* spores as described above. 12 hours later, cDC were assayed for maturation by flow cytometry.

### Detection of IFNa, IFNg and IL-12p40

IFNa was measured in supernatants from *in vitro* cultures of young or aged mouse pDC after *E. cuniculi* stimulation (PBL Biomedical Laboratories) by ELISA according to manufacturers instructions. IFNg was measured in supernatants from *ex vivo* cultures of DC adoptive transfer experiments and IL-12p40 was measured in supernatants from *in vitro* DC co cultures by ELISA according to manufacturers instructions (ELISA Max Standard Set, eBioscience).

### Flow cytometry staining and antibodies

Splenocytes from recipient mice and cells from in vitro cultures were first stained for viability in 1× PBS using Live/Dead Fixable Amine reactive dye (Invitrogen). After washing 2× with 1× PBS, surface staining was performed using stain wash buffer (SWB, 2% FBS in 1× PBS and EDTA) for 20 minutes on ice. For DC maturation assays, sort purified cells were stained for CD40, CD80, CD86, ICOSL, PD-L1, PD1, CD40L, MHC Class II, CD11c, B220, and GR1 (eBioscience). Apoptosis was measured using active caspase-3 kit (BD biosciences). For T cell activation, cells were surface stained with CD25 and CD8 (eBioscience). For intracellular staining, T cells were fixed and permeabilized for 45 minutes on ice (BD bioscience, Fix/Perm solution) followed by intracellular staining in permeabilization wash buffer with anti-IFNG antibody (BD Biosciences) for 1 hour on ice. Cells were then acquired using FACs Aria (BD Biosciences) first gating on live cells then excluding clusters. All samples analyzed with FlowJo software (Tree Star).

### Statistical analysis

Statistical analysis of the data was performed using Graphpad Prism 5 software with either two-tailed Student's *t* test or 1 way ANOVA and post analysis with Bonferroni's multiple comparison test. Statistical significance is indicated as * with *p*<0.05, unless otherwise noted.

## Supporting Information

Figure S1 **pDC depletion** ***in vivo*** **.** Naïve 8 week old C57BL/6 mice were depleted of pDC by i.p. injection of 150 ug/ml of 120G8 pDC specific antibody. Spleens were harvested 12 hours later and single cell suspensions were analyzed by flow cytometry pDC (CD11c lo GR1+B220+, top right quadrant in dot plots). (A) Representative dot plots of CD11c-lo-med splenocytes stained for B220 and GR1 from isotype (right) and 120G8 (left) treated mice. Dot plots are from 1 experiment that was repeated separately twice.
(EPS)

Figure S2 **Diphtheria toxin treatment of CD11c DTR-EGFP transgenic mice depletes DCs.** Naïve 8 week old CD11c DTR-EGFP mice were treated with 1 dose of 100 ng DT i.p. and 6 hours later splenocytes harvested and single cell suspension made. Splenocytes were then assayed for cDC (CD11c Hi GFP+) by flow cytometry. (A) Representative dot plots show CD11c hi GFP+ splenocytes (top right quadrant) from PBS (right) and DT (left) treated mice. Dot plots are from 1 experiment that was repeated separately twice.
(EPS)

Figure S3 **CD8 T cell activation by re-sorted cDC** ***in vivo*** **is dose dependent.** Sort purified naïve young cDC were stimulated with irradiated *E. cuniculi* spores for 12 hours then resorted. cDC viability was measured prior and post re-sorting using fixable Live/Dead staining. After resorting, cDC were adoptively transferred at different cell numbers; high (4.0×10^5^), medium (2.5×10^5^) or low (5.0×10^4^) cell numbers to DC depleted CD11c DTR transgenic mice (DT 100 ng, 6 hours). 4 days later, activated CD25+ CD8+ T cells were assayed for IFNg expression by flow cytometry. (A) Dot plots show frequency of activated CD25+ CD8+ T cells that are IFNg+. (B) Bar graph shows the mean ± SD of activated CD25+ CD8+ T cells that are IFNg+ from mice receiving different doses of resorted cDC. (C) Bar graph shows the IFNg MFI of cytokine producing CD25+CD8+ T cells from recipient mice. (D) Dot plots show the cDC viability prior to (left panel) and post re-sorting (right panel) by Live/Dead staining. Data is representative 1 of 2 experiments repeated at separate times with an n = 4 mice/group per experiment. *p<0.05 by Student's *t* test.
(EPS)

Figure S4 **Aged cDC are unable to efficiently mature after** ***E. cuniculi*** **infection.** 1.25×10^5^ Sort purified splenic cDC from naïve aged (12 month) or young (8 week) mice were cultured alone or co-cultured with 1.0×10^4^ naïve young or aged mouse pDC and stimulated with irradiated *E. cuniculi* spores for 12 hours. Aged or young cDC cultured alone (A and B), aged or young cDC co-cultured with young pDC (C and D) and aged cDC co-cultured with either young or aged pDC (E and F) were then analyzed for CD86 and MHC Class II expression after live/dead staining and exclusion of cell clusters. Histograms show CD86 (A,C and E) and MHC Class II (B,D and F) levels on cDC from each culture condition. Bar graphs corresponding to (A,C, and E) CD86 and (B, D and F) MHC Class II on cDC for each culture condition show the mean ± SD of MFIs from 2 independently repeated experiments. * Denotes significance measured by 1 way ANOVA and Bonferroni's multiple comparison test with p<0.05.
(EPS)
